# Syphilis: A Case in Practice

**Published:** 1901-01-15

**Authors:** L. M. James


### SYPHILIS: A CASE IN PRACTICE.

BY L. M. JAMES, D.D.S.

This paper was read at the April, 1900, meeting of the Washtenaw County Dental Association, and it was with some reluctance that I undertook its preparation, not because I believed the case would be lacking in interest to members of the dental profession, but because there were constitutional conditions involved and methods of treatment indicated that required a much broader knowledge of certain pathological subjects than I possess. Then, too, we do not usually take great pleasure in reporting our failures, for I am compelled to admit that this case *was* a failure, though the conditions were such that a favorable termination was probably impossible. I did not at that time, nor do I intend now, to enter upon any elaborate analysis of this case. I shall simply place the facts before you, trusting that some of the more experienced members of the profession may be able to suggest methods of treatment that would lead to more successful results.

Some months ago, a patient whose teeth had been under my care for several years, came to the office and stated that one of her front teeth had for several days been giving her much trouble. On examination, I found the upper, left, central incisor a perfectly sound, well-formed tooth—very loose and very sensitive and the surrounding tissues very much inflamed. The outer or labial margin of the alveolus was somewhat absorbed and the gum much swollen and almost purple in appearance. The tissues about the palatal portion of the neck of the tooth were apparently in a better condition, though there was a slight swelling about one-half inch back of the tooth. The pulp was evidently dead and the left lateral and right central showed marked symptoms of periosteal inflammation. The patient's health seemed generally good, though at the time she seemed to have a slight cold, and the lining membrane of the left nostril, especially, I found to be slightly congested. I concluded that the condition of the tooth or teeth was the result of a blow, for we often meet with such cases. At first, the patient could not recall anything of the kind, but afterward remembered that some six or seven weeks before the first symptoms of trouble appeared, while preparing kindling, a piece of the wood glancing from the ax gave her quite a sharp blow on the mouth. The pain was quite severe at first, but passing away after a few minutes she had forgotten all about the occurrence.

Believing that my diagnosis was correct, I proceeded to treat the case accordingly. For some time the tooth was so very sensitive that the necessary opening into the pulp chamber could not be made without causing very severe pain, but after an application of cocain solution an incision was made through the labial surface of the gum and process to the apex of the root and also through the swelling in the palate. The alveolar plate between the apex of the root and the gum I found so nearly absorbed that but little effort was required to penetrate it.

Although there was very little discharge through either of these openings, the extreme soreness in the central disappeared after a few days and I was then able to drill through the palatal surface into the pulp chamber. After cleansing the canal I found that a solution of pyrozone pumped into this opening passed out freely at those first made, with but little effervercence, however.

I now supposed the case was well in hand, but after some time spent in further treatment I found that there was not only no improvement, but that there was a very decided change for the worse. The tooth became so loose that I feared it would drop from the socket; the two adjoining teeth became more troublesome; the membrane about the opening in the palate assumed a decidedly ugly look and there were evidences of necrosis of the bone, while the absorption of the labial portion of the alveolar process extended over the left lateral and right central. And still more alarming, an injection through the palatal opening passed upward and was discharged through the left nostril, each treatment being followed by considerable hemorrhage. Dr. Watling saw the case frequently, and at his suggestion I finally used as a wash a weak solution of ferrous sulphate, cleansing the canals thoroughly with this once or twice daily. At first this seemed to produce some slight improvement, but there being no marked change and having already removed several portions of necrosed bone from the palate, I finally extracted this tooth and also the two adjoining teeth, these being almost as loose and sensitive as was the left central when I first saw the case. But even then the tissues did not heal, more necrosed bone was removed, both from the palate and the sockets of the teeth extracted, and after some days a surgeon was called to examine the case. He also was puzzled and for some time refused to take the patient from my hands, though he suggested, what I confess I had not for an instant suspected, that there were syphilitic indications. The patient after a time—by her mother's advice, I afterward learned—decided to consult the physician who had attended at her birth and—the problem was solved. The case was clearly hereditary syphilis.

The Doctor informed me that at the child's birth and during the period of gestation as well, the mother was afflicted with syphilis in one of its worst forms, the disease being contracted from the father. For many years, the patient is now about thirty, he had watched for symptoms of the disease in the offspring, believing that sooner or later it must appear. He immediately took charge of the case and after a long period of constitutional treatment succeeded in relieving the trouble and had he lived, for he was a very able man, would probably have effected a cure. But unfortunately for the poor girl and for us all, death removed the good old Doctor from among us and the patient, poor victim of a father's sin, knowing the cause of her terrible misfortune, for weeks refused to consult another physician. At last, however, she consented to receive further treatment and applied for relief to the doctor who first examined her. He now assures me that a cure is possible, but that constant care will be required for two or three years to prevent a return of the disease. When I last saw the case the mouth seemed to be entirely healed but there was a very marked depression at the bridge of the nose, showing that the bones of that region had suffered considerably. The loss of the teeth has not yet been remedied, for it is feared that at present even the slight irritation of an artificial substitute may result disastrously to the remaining teeth or the tissues of the mouth.

What more is there to say? Could I have saved those teeth had I known earlier the cause of the disturbance? Should I have at once recognized the cause? The symptoms of syphilis were not well marked; the teeth were well formed; the soft palate in a perfectly healthy state. There was no apparent odor from the discharge. I had never, in the years I had cared for the teeth of this patient, seen the slightest indication of a syphilitic diathesis. The first physician, an able practitioner, was puzzled, though the condition of the nose led him to suspect the truth. The girl herself was above suspicion and the parents I had never known. I *know* that the tooth first affected was beyond help, and I very much doubt if the other two could have been saved, even though constitutional treatment had been resorted to from the first.

The father, whose sins are thus visited upon his child, has for many miserable years been an inmate of an asylum for the insane. A just punishment, is it not?

[At a meeting of the Washtenaw County Dental Society, December 10th, Dr. James reported that this case was in a very hopeful condition. The local trouble had disappeared and the tissues seemed normal. The patient is wearing a plate with satisfaction. The physician pronounces the case cured but will keep close watch over it, and continue systemic treatment for a year or so.—Ed.]

